# Searching for empirical evidence on traffic equilibrium

**DOI:** 10.1371/journal.pone.0196997

**Published:** 2018-05-07

**Authors:** Mehmet Yildirimoglu, Osman Kahraman

**Affiliations:** 1 School of Civil Engineering, The University of Queensland, St Lucia, QLD, 4072, Australia; 2 Department of Physics & Astronomy, University of Southern California, Los Angeles, CA, 90089, United States of America; 3 R&D Center, Arcelik A.S., Tuzla, Istanbul, 34950, Turkey; Beihang University, CHINA

## Abstract

Cities around the world are inundated by cars and suffer traffic congestion that results in excess delays, reduced safety and environmental pollution. The interplay between road infrastructure and travel choices defines the level and the spatio-temporal extent of congestion. Given the existing infrastructure, understanding how the route choice decisions are made and how travellers interact with each other is a crucial first step in mitigating traffic congestion. This is a problem with fundamental importance, as it has implications for other limited supply systems where agents compete for resources and reach an equilibrium. Here, we observe the route choice decisions and the traffic conditions through an extensive data set of GPS trajectories. We compare the actual paths followed by travellers to those implied by equilibrium conditions (i) at a microscopic scale, where we focus on individual path similarities, and (ii) at a macroscopic scale, where we perform network-level comparison of the traffic loads. We present that non-cooperative or selfish equilibrium replicates the actual traffic (to a certain extent) at the macroscopic scale, while the majority of individual decisions cannot be reproduced by neither selfish nor cooperative equilibrium models.

## Introduction

Our lives are trains of decisions, many are made individually in a matter of seconds as in changing lanes in busy traffic [1], some others are crafted as carefully as possible to harvest the best of outcomes despite a deceptive lack of a clear foresight of the future and may involve collectives of people of varying size, as in choosing a place to set up a firm [2]. The dynamics of how these decisions come together and interact with each other within a resource limited setting is what fuels and shapes our languages [3], markets [4], and social lives [5]. In line with such a fundamental importance, a great deal of intellectual effort has been devoted to elucidate the principles governing decision making at various scales, giving birth to many now established disciplines such as rational choice theory [6], game theory [7], as well as relatively recent fields including sociodynamics [8, 9], behavioural economics [10] and algorithmic game theory [11]. Leveraged with the massive amounts of data on our social and economic actions being gathered with increasing accuracy thanks to the widespread access to information and communication technologies, such theoretical approaches hold the promise of addressing many challenges our modern societies face today, including the questions of sustainability and efficiency of our cities under their rapid urbanization.

With the spatial and social proximity of dense urban environments tightly entangling the decisions made by civil agents such as individuals, firms and policy makers, cities provide a paradigmatic scene of decision dynamics, incubating social, economic, and environmental consequences at the global scale as they are now home to the majority of world population [12]. In view of the appreciation of cities as complex systems emerging from the bottom up organization of local interactions [13, 14], a toolbox of data-driven analytic approaches drawing on a wide range of disciplines ranging from physics to computer science and statistics are now being compiled [15] in an attempt to better understand urban processes and provide planning strategies to limit their unintended consequences. One such critical process plaguing cities with congestion and pollution, mobility on urban road networks, has been particularly challenging to understand due to the complex interaction between road infrastructure resulting from studied decisions of policy makers and traffic conditions emerging from travel choices of users, such as departure time and route choices. The mainstream understanding on the complex interplay between the supply provided by road infrastructure and the demand generated by travel plans of drivers is built upon the contrast between two idealized traffic flow states, originally defined by Wardrop [16]. While user equilibrium (UE) state corresponds to conditions in which drivers choose routes to selfishly minimize their travel times and costs (i.e. Nash equilibrium applied on traffic networks), in system optimal (SO) state, drivers act cooperatively, as if governed by the rule of a benevolent dictator, to ensure global optimality by minimizing aggregated travel times over all network. The efficiency loss arising from settling for UE assignment rather than SO, or for selfish rather than social equilibrium, is hence sometimes dubbed as the price of anarchy [17, 18].

While UE assumes that travellers are omniscient with the perfect knowledge of travel costs along the network at their disposal and that they rationally choose the routes that minimize their travel costs, stochastic user equilibrium (SUE) acknowledges that travellers might not be fully informed about network conditions, and therefore choose the routes that minimize their perceived travel costs [19]. There is a vast literature of discrete choice models developed for modelling the perception error and reaching SUE conditions [20–25]. Apart from deterministic and stochastic equilibrium definitions, a large body of literature focuses on bounded rationality to address the lack of accurate information and to model the inertia to switch decisions [26–28]. Nevertheless, given that the deterministic UE conditions can be established by virtually parameter-free algorithms and are empirically supported in controlled settings [29, 30], they form the basis of many traffic assignment models, which are essential to estimate travel demand under hypothetical scenarios [31].

However, it still remains unclear how close the behaviour of drivers is to UE in realistic settings. Despite the abundance of studies on finding traffic equilibrium in transportation and urban planning literature, only few studies focus on the empirical validation of equilibrium principles. Refs. [32–34], using limited GPS records, refs. [35, 36], using survey data, and ref. [37], using lab experiments, show that in practice most drivers do not choose shortest path and comply with deterministic user equilibrium conditions. Nevertheless, these studies do not incorporate big data sources into their models, and they test the equilibrium assumptions only from a user perspective, i.e. similarity between actual and shortest path at the individual level. While congestion seems unavoidable, drivers increasingly take advantage of real-time information through GPS devices and smart phones, which in turn generates passive and disaggregated path observations. These observations, when put together, can provide valuable information on how traffic is organized at the network level and aggregated patterns emerge from the interactions between users.

In this work, we use real traffic data to obtain travel demand and traffic conditions across Shenzhen, a busy city in China, and we look for the signature of the two fundamental equilibrium conditions, *i.e.* UE and SO, both in the behaviour of individual drivers and in the aggregate organization of travel choices at the road network level. To generate travel demand, we begin by mining massive GPS records from taxis. Using the taxi trips with passengers, we identify the origin and the destination locations within the city. We parse the publicly available OpenStreetMap data and build the road network graph. OpenStreetMap provides the physical distance across the network and the category of roads which allows us to calculate free flow travel times. To determine actual traffic conditions, we process the taxi trajectories and estimate link travel times. Using travel choices and traffic conditions derived from real traffic data, we finally explore the relationship between the actual paths followed by travellers to those implied by UE and SO equilibrium conditions at two different scales: (i) at the microscopic scale, we focus on the user perspective and we compare individual path similarities and (ii) at the macroscopic scale, we perform network-level comparison of the traffic loads for the entire road infrastructure. The findings from this multiscale comparison reveal a strong contrast between different representations of travel choices. Considered from the individual user perspective, actual travel choices appear to differ remarkably from those associated to deterministic equilibrium conditions, while network-level analysis presents significant correspondence between the actual choices and equilibrium choices, in particular for UE. More generally, our analysis shows that decisions by collectives of agents in a resource limited environment may organize into diverging patterns at different scales and our results has thus implications not only for urban mobility but also for other biological and social systems where agents compete and cooperate for resources.

## Results

Traffic equilibrium models concern the selection of routes given the demand between origins and destinations in transportation networks, and they can be formulated as variational inequality, nonlinear complementarity or fixed-point problems [38, 39]. In fact, these models present a system of nonlinear equations, and the most efficient solution approach would be Newton’s method which is based on replacing the nonlinear function with its first-order approximation. However, traffic simulation tools, which are becoming increasingly popular, involve a large number of parameters and do not have an analytical form. Therefore, the first-order derivatives are usually not available in the traffic equilibrium context. Fixed-point formulations, which do not require the computation of derivatives, are deemed the most efficient approach to establish equilibrium conditions.

Solutions to both selfish or social equilibrium can be found through fixed-point formulations and iterative mechanisms, with each agent selecting its route given the traffic conditions that result from the choices of the others in the preceding time periods and in the previous iterations. A fixed-point problem is based on a continuous function *f*: *X* → *X*, and a fixed-point solution is x* ∈ *X* such that *f*(x*) = x*. Fixed point problems are traditionally solved with iterative methods that build on a starting point *x*_0_ and a succession of points of the form;

$$\begin{array}{c} x_{k+1}=x_{k}+\alpha_{k}*\left(f\left(x_{k}\right)-x_{k}\right) \end{array}$$ (1)

where *α*_*k*_ ∈]0, 1]. Note that when *α*_*k*_ = 1/*k*, the method becomes the well-known method of successive averages (MSA). On the other hand, when *α*_*k*_ = 1, the output of one iteration becomes input to the next *x*_*k*+1_ = *f*(*x*_*k*_) and the scheme is referred to as Picard iteration [40]. MSA is commonly used in the transportation context assuming that it helps the algorithm to converge (note that it inherently converges to a stable point with decreasing 1/*k* values). However, Picard iteration should converge to the same point, in case a unique solution exists to the fixed-point problem.

**Fig 1:**
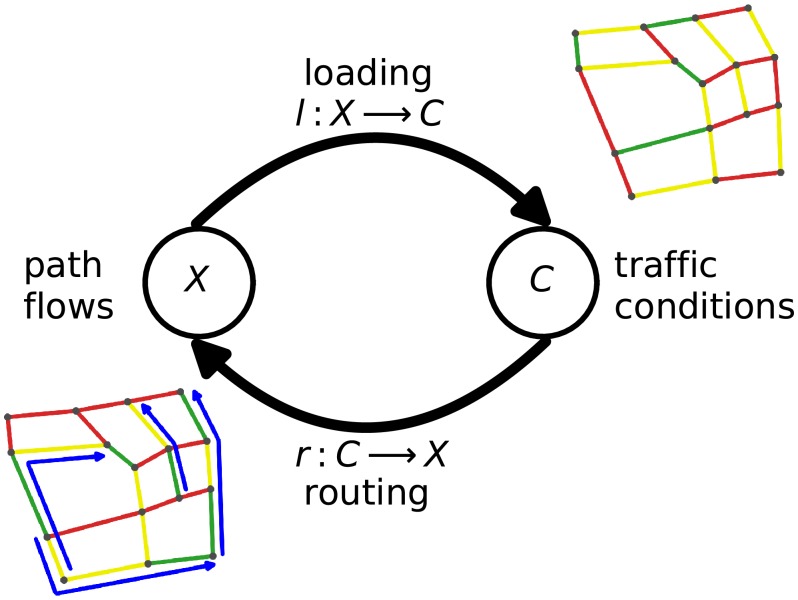
Illustration of finding the equilibrium solution in traffic as a fixed-point iteration. The equilibrium solution of path flows satisfies *f*(*X*) = *X*, where *f* = *r* ∘ *l* is joint process of network loading *l* : *X* → *C* that generates traffic conditions *C* based on flows *X*, and routing *r* : *C* → *X* that assigns path flows given traffic conditions *C*.

In our problem, *X* denotes the path flows, and *f* represents the joint process of network loading, i.e. *l* : *X* → *C*, that produces traffic conditions *C* and route assignment, i.e. *r* : *C* → *X*, that in turn builds path flows *X* (see Fig 1 for the illustration of a single iteration). The computation starts with an initialisation step, i.e. *x*_0_, where certain assumptions are needed (e.g., empty network, free-flow traffic conditions), and continues until the variables reach a predefined level of convergence. Details about the data, travel time estimation and the methodology are presented in the Materials and Methods section.

In this study, we do not aim to design a function *f* which will yield the stable point x* starting from an initial point *x*_0_. In other words, our goal is not to establish equilibrium conditions starting from an empty network and using a given demand profile. In contrast, we observe the existing path flows *x*_*k*_ ∈ *X* and traffic conditions *c*_*k*_ ∈ *C* in the last iteration *k* through an extensive GPS data set, and we test the route assignment process, *r*, which is expected to produce similar path flows at the (hypothetical) equilibrium point, i.e. *x*_*k*_ ≈ *x*_*k*+1_ ≈ x*. Particularly, we assume the observed path flows *x*_*k*_ ∈ *X* and traffic conditions *c*_*k*_ ∈ *C* corresponding to the last iteration *k* of the fixed-point formulation represent the equilibrium solution to the problem. Therefore, an additional Picard iteration should not significantly deviate from the existing values. Given the observed traffic conditions, the deviation between observed and estimated path flows is expected to provide a measure of closeness to the equilibrium state. Although not explicitly stated, [34] relies on the same mechanism to test Wardrop’s first principle and reveals the route choice characteristics (i.e. the similarity between observed and shortest paths) as a whole. Note that, in this study, we do not develop a network loading function *l* either, because we rely on real traffic conditions that can be estimated through GPS records. In a complete traffic equilibrium model, this step is usually accomplished through traffic simulation tools that require tedious calibration adjustments and complete demand profile (we only have partial demand information from the vehicles equipped with GPS devices). As we are only interested in the last iteration of the fixed-point formulation and the current route choices have already been evaluated with real traffic dynamics, a loading function that mimics the real world dynamics seems unnecessary.

**Fig 2:**
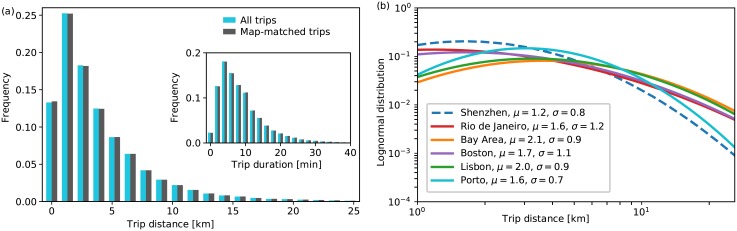
Trip distance and time distributions. (a) Comparison of trip distance distributions for all trips (cyan) and for map-matched trips (dark grey). Inset compares trip duration distributions for all trips (cyan) and for map-matched trips (dark grey). (b) Comparison of the lognormal distribution fitted to trip distances in Shenzhen to the lognormal distribution of commuting trip distances in five major cities as reported in Ref. [41].

We base our analysis on GPS traces of taxis collected on a weekday in Shenzhen, China (see *Data* section in Materials and methods). Given that GPS traces correspond to geographic coordinates, we first apply a map-matching algorithm (see *Map matching* section in Materials and methods) to identify the paths that vehicles actually follow in the network that we extracted from OpenStreetMap (see *Road network* section in Materials and methods). Fig 2(a) introduces the distribution of trip distance and duration for all the trips and the subset of map-matched trips (approximately 20% of the trips have been removed after map-matching due to the lack of feasible paths). We do not observe a significant difference between the two sets in terms of distance and duration distribution, which implies that the map-matching process does not create a bias in our analysis. From Fig 2(a), we see that most trips span a distance less than 10 km, and there are very few trips that cross more than 20 km. Similarly, we see that most taxi trips take less than 15 min, and only few exceed 30 min (Fig 2, inset). Fig 2(b) presents the lognormal distribution that trip distances follow in Shenzhen and compares it with five cities studied in [41], where cell phone tower data are processed to estimate trip distances. The resulting distributions have distinct features as each city is unique in terms of size, population and density. While our analysis is limited by a rather small area (approximately 350 km^2^) that taxis serve in Shenzhen, ref. [41] focuses on metropolitan areas whose size ranges from 2,000 km^2^ to 18,000 km^2^. This is the main reason for a relatively low density value corresponding to high distances in Shenzhen. Despite all differences, trip distance distribution in Shenzhen shares similar characteristics with other cities; majority of trips cover relatively short distances and few trips go beyond 20-25 km. As our data set only represents taxi trips with passengers, one may expect a different distance/duration distribution for all vehicles. In particular, there may be more trips with longer distance/duration in the actual distribution; however, as these long trips are expected to constitute a very small portion among all trips, we do not expect significant changes in our results.

The results from our analysis will be presented in two sections; user-perspective and network-perspective. In the user perspective section, we focus on the comparison between the observed route and the shortest path for each trip separately, compute a spatial similarity score and present the distribution of the resulting variables across all trips. Even though the results are aggregated to produce the distributions, the raw variables (e.g., spatial similarity, whether or not a route is equivalent to its shortest path) indicate the similarity between the equilibrium state and existing conditions from a user perspective. On the other hand, the network perspective undertakes the comparison of node loading resulting from the observed routes and the equilibrium routes. As we turn our attention from user trips to network components, the second section is named ‘network-perspective’. To the best of our knowledge, this work is the first attempt to investigate the aggregated traffic loads that result from widely assumed behavior patterns. Aggregated node or link flows have been largely employed for calibration purposes; however, these methods rely on observations from a subset of network components and look to adjust the model parameters to match them. The link between behavioral aspect and network loads, which we explore in this study, has not been considered before. Additionally, the literature on route choice behavior relies on limited data sets that do not allow statistically significant estimates of network loads. Hence, they focus on user-level or trip-based comparisons. In contrast, the large-scale GPS data set, which is employed in this article, allows us to observe traffic conditions in most part of the network (excluding the local roads that are not frequently being used) and compute (partial) traffic loads on the network nodes. Note that as we only observe the vehicles that are equipped with GPS devices, we estimate partial loads on the network nodes. Nevertheless, since the same trip set is being used for the calculation of observed and estimated traffic loads and the fixed-point formulation remains valid for any subset of trips, our calculations are not affected by any mathematical bias.

### User perspective

**Fig 3:**
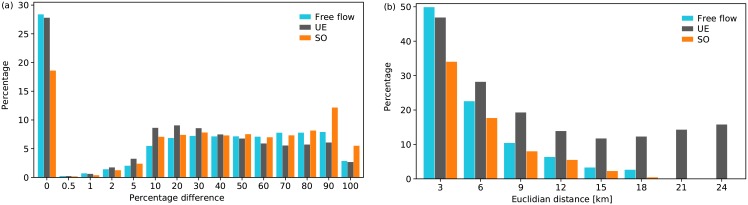
Difference between GPS-revealed actual routes and the shortest paths. (a) Overlap between actual routes followed by the users and shortest paths resulting from travel costs computed following FF (cyan), UE (dark grey), and SO (orange) assumptions. (b) Percentage of users following the shortest path computed according to FF (cyan), UE (dark grey), and SO (orange) assumptions grouped by Euclidean distance between the origin and the destination of the trips. We used a threshold of <10% on route overlaps to classify a user to follow the shortest path.

In this section, we compare the map-matched routes of travellers to the shortest paths implied by different route choice scenarios in terms of overlap percentages. To determine the shortest path, we use three types of travel cost; free flow, experienced and marginal travel times that correspond to free flow (FF), UE and SO patterns, respectively (see *Travel time estimation* section in Materials and methods). Even though FF does not represent a state of equilibrium, it is included here as a benchmark scenario that illustrates a naive loading mechanism with no choice coupling between travellers. Fig 3(a) presents route overlaps between the actual routes and the shortest paths resulting from the above travel costs. If two routes completely overlap, the difference should be 0. If they do not overlap at all, the difference should be 100%. Using the most strict overlap definition (i.e. 0% threshold), we observe that around 28%, 28% and 18% of paths are in full compliance with shortest paths resulting from FF, UE and SO assumptions, respectively. Considering 10% threshold to assume two paths are identical, this value goes up to 37.6%, 38.2% and 26.3% for the three cost definitions in the same order. While FF and UE patterns produce close values and similar distributions throughout the range, SO consistently produces less paths with low dissimilarity and more paths with high dissimilarity. Although the best matching performance is achieved with FF and UE patterns, we note that neither of the cost definitions is able to reproduce the observed patterns to a large extent at the level of individual trips.

The number of alternative routes, and thereby route choice behaviour is not the same for all origin and destination pairs. For example, if the trip covers a short distance, the entire path could consist of one straight road and even the second shortest path would be much longer. On the other hand, if the distance is very long, the common strategy would be to reach the nearest freeway connection and get off at the nearest interchange to the destination point, which would imply a very high overlap percentage among shortest paths. In order to investigate the relation between trip distance and overlap percentage, we have divided the data set into groups with respect to Euclidean distance between origin and destination points. Fig 3(b) presents the percentage of routes that are equivalent to shortest paths based on FF, UE and SO conditions in each distance group. We classify routes that differentiate from the shortest path by less than 10% in length as those following the shortest time path. As expected, travellers are more likely to follow the shortest path when the trip is short; all travel cost definitions perform the best in the lowest distance group. Nevertheless, FF and UE patterns significantly outperform SO within this group, which implies that social equilibrium would require the travellers to take significant detours even for short distance trips. We also observe that the matching performance of FF routes drops very fast as the trip distance increases. On the other hand, UE converge to a value around 15% with increasing distance. Our expectation regarding long distance trips seems not to be valid for most of the observed routes.

**Fig 4:**
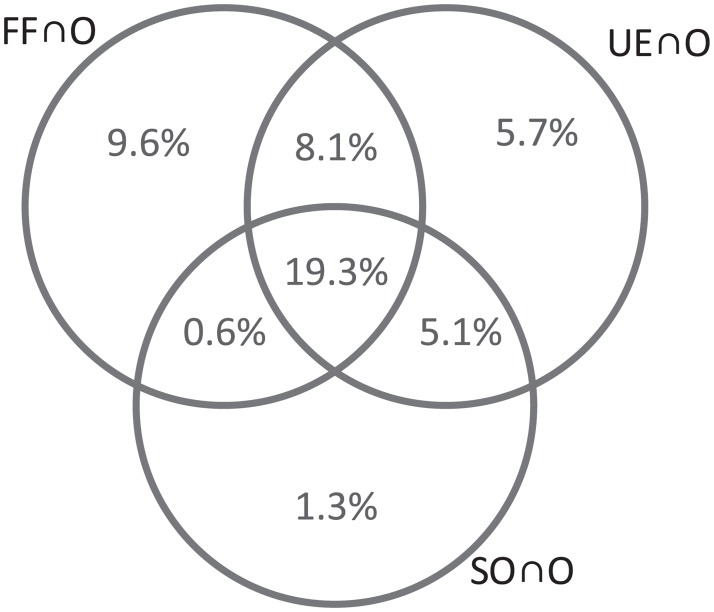
Venn diagram depicting the relations between the hypothetical patterns *FF* ∩ *O*, *UE* ∩ *O* and *SO* ∩ *O* represent the intersection between the observed paths *O* and the three hypothetical patterns; FF, UE and SO, respectively. The numbers indicate the percentage of correctly reproduced paths in each region.

To further analyse the correspondence between the hypothetical patterns, we identify the paths that are correctly reproduced (within 10% margin) by three hypothetical patterns, build three sets (i.e. *FF* ∩ *O*, *UE* ∩ *O*, *SO* ∩ *O*, which represent the intersection between the observed paths *O* and the three hypothetical patterns) and determine all possible logical relations between them. Fig 4 presents a Venn diagram that depicts the relations between the three sets and includes the percentage of correctly reproduced paths in each region. Note that the sum of all elements in *FF* ∩ *O*, *UE* ∩ *O* and *SO* ∩ *O* lead to 37.6%, 38.2% and 26.3%, respectively, as reported before. Surprisingly, 19.3% of the observed paths are reproduced by all three hypothetical patterns, which indicates a high degree of similarity between them. Further, we note a strong accordance between FF and UE; the intersection region, (*FF* ∩ *O*) ∩ (*UE* ∩ *O*), constitutes more than 70% of each set. In overall, this analysis presents no clear dominance of one pattern over the other, particularly UE over FF.

Our findings are in line with previous studies [34–36]; even with an overlap tolerance, not more than 35% of trips follow the shortest path based on any cost definition. Nevertheless, this observation does not fully explain “How far is traffic from equilibrium?”. There is a need for a novel approach from network perspective that investigates aggregated patterns resulting from the equilibrium assumptions. The next section intends to address this gap.

### Network perspective

**Fig 5:**
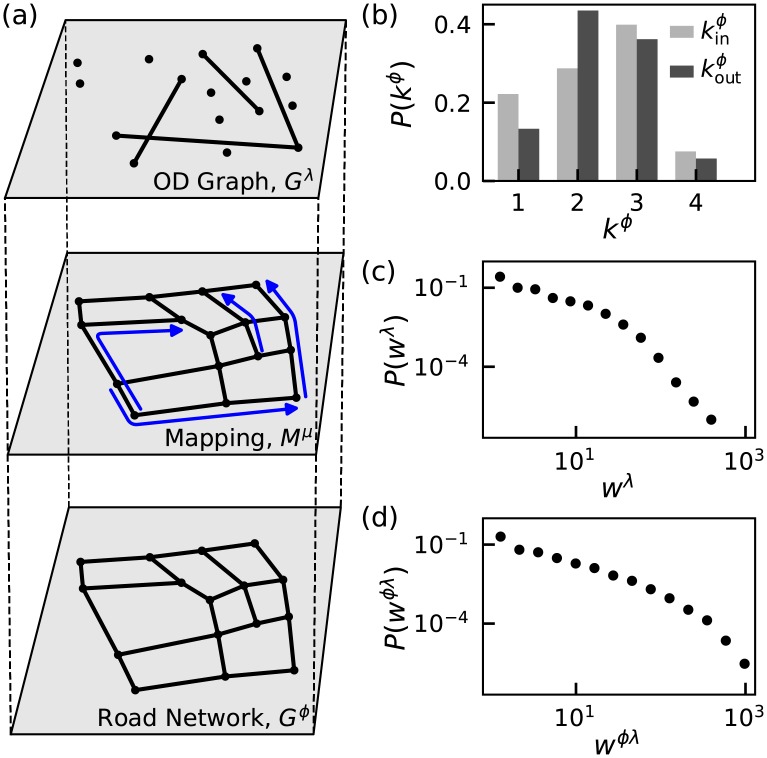
Description of the multilayered network approach. (a) Illustration of the two-layer network; the logical OD graph and the physical road network. Each logical edge from the OD graph is mapped onto the road network as a path in the mapping layer. (b) Node degree distribution of the physical layer. (c) Total weight distribution of nodes in the logical layer *G*^λ^. (d) Weight distribution of edges obtained from the mapping *M*^*μ*^ using actual taxi trajectories.

Having established the performance of FF, UE and SO route choice scenarios to capture the behaviour of individual drivers, in this section, we adopt a coarse-grained approach and study the network-level traffic decision patterns resulting from aforementioned route choice scenarios. Due to complex organization of mobility patterns in relation to road infrastructure, simple network representations of transportation systems are far from providing a comprehensive characterization of such systems within the framework of complex spatial networks [42]. To disentangle the network of traffic flows from the underlying road infrastructure, here we follow Ref. [43] and use a two-layered network model with one physical and one logical layer. As illustrated in Fig 5(a), while the lower-layer physical graph *G*^*ϕ*^ = (*V*^*ϕ*^, *E*^*ϕ*^) represents the physical road network with nodes *V*^*ϕ*^ ≡ *V* and edges *E*^*ϕ*^ ≡ *E*, the upper-layer logical graph *G*^λ^ = (*V*^λ^, *E*^λ^) captures the traffic over the physical graph with nodes *V*^λ^ ≡ *V*^*ϕ*^ and edges *e*^λ^ = (*u*, *v*) pairing the origin *u* and the destination *v* of traffic flows. In our case, both the physical and logical layers are directed and weighted graphs. Fig 5(b) shows node degrees $k_{\text{in}}^{\phi }$ and $k_{\text{out}}^{\phi }$ associated with, respectively, incoming and outgoing edges of the physical graph *G*^*ϕ*^. Consistent with previous studies of urban road network topology [44, 45], we find that most nodes have a degree ≤4, with a cumulative probability *P*(*k*^*ϕ*^ ≥ 5) < 0.01 and the average degree 〈*k*^*ϕ*^〉 ≈ 2.32 both for incoming and outgoing edges. Fig 5(c) shows that the distribution of node weights of the logical graph *G*^λ^ is highly right-skewed indicating that while a large portion of nodes has low weights (< 10), few nodes bear very large weights (> 100).

Each logical edge *e*^λ^ is mapped onto the physical graph as a path *M*^*μ*^(*e*^λ^) navigating the traffic from *u* to *v*. This mapping may be given explicitly as the actual paths constructed from GPS tracking or may be defined implicitly through shortest paths. Fig 5(d) shows that edge weights obtained following the former scenario with actual paths obtained from GPS tracks have a right-skewed distribution similar to the distribution of logical weights shown in Fig 5(c). Finally, load *l* of a node *u*^*ϕ*^, corresponding to the amount of traffic flowing through *u*^*ϕ*^, is defined as the sum of the weights of logical edges whose paths *M*^*μ*^(*e*^λ^) cross *u*^*ϕ*^.

$$\begin{array}{c} l\left(u^{\phi }\right)=\sum_{e^{\lambda }:u^{\phi }\in M^{\mu }\left(e^{\lambda }\right)}w\left(e^{\lambda }\right). \end{array}$$ (2)

**Fig 6:**
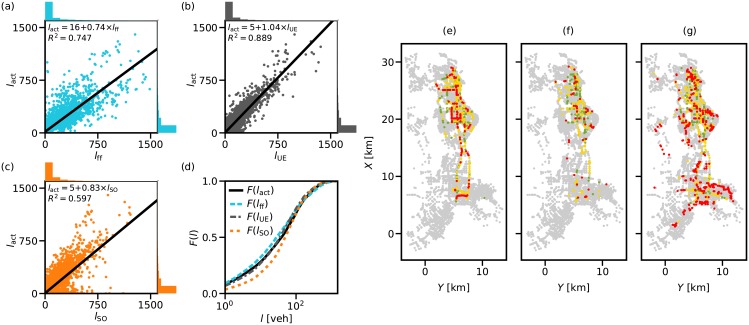
Comparison in node loads between actual routes and shortest paths. Comparison of the node loads *l*_*act*_ that result from the actual routes as revealed by GPS tracks and map-matching process to the node loads resulting from shortest paths based on (a) the free flow travel time *l*_*ff*_, (b) the experienced travel time *l*_*ue*_, and (c) the marginal travel time *l*_*so*_. (d) Cumulative distribution of the resulting traffic loads; *F*(*l*). Spatial distribution of errors with (e) FF, (f) UE and (g) SO patterns. We include nodes with negligible estimation error (gray) and nodes with absolute percentage error <50% (green), >50% and <100% (yellow), >100% (red).

In Fig 6(a)–6(c), we compare the traffic loads (or node loads) that result from the actual routes as revealed by GPS tracks and map-matching process (i.e. *l*_act_) and the ones that are associated with shortest paths based on the free flow travel time (i.e. *l*_ff_), the experienced travel time (i.e. *l*_UE_) and the marginal travel time (i.e. *l*_SO_), respectively. We observe varying levels of proportionality between the actual and the estimated loads across the three cases. However, we note a significantly stronger fit with UE patterns than with the other two. In Fig 6(a) and 6(c), we see that the scatter or the mismatch between the observed and estimated traffic loads becomes more evident with increasing *l*_act_, which indicates poorer estimation quality for high load carrying components. On the other hand, the scatter seems to be homogeneous over the entire range of *l*_act_ in Fig 6(b).

To further analyse the estimation results, we fit a linear function of the form *l*_act_ = a*l_*x*_ + *b*, where *l*_*x*_ is *l*_ff_, *l*_UE_ or *l*_SO_. Regression lines are introduced in Fig 6(a)–6(c), while the coefficient estimates and the output statistics are presented in Table 1. In overall, FF and SO assumptions cause overestimation of traffic loads in the network (*a* is significantly below 1), while UE produces compatible and well matched results (*a* being very close to 1). Considering the regression coefficients, the fit quality (i.e. R^2^ and Adj.R^2^), F-statistics and the error measures (i.e. MAE and RMSE), we can confidently conclude that, although traffic in reality is not a perfect UE, it is much closer to UE than FF or S0. Note that *p* values (for the F-test on the model) indicate a significant linear regression relationship at 0.01 level between the response variable and the predictor variables in all three cases.

**Table 1:** Regression results and error measures.

	Estimates							
	*a*	*b*	R^2^	Adj.R^2^	F(10^4^)	*p*	MAE	RMSE
(FF)	0.74*	16*	0.75	0.75	2.96	0*	27	74
(UE)	1.04*	5*	0.89	0.89	8.05	0*	19	43
(SO)	0.83*	5*	0.60	0.60	1.49	0*	41	83

MAE: mean absolue error, RMSE: root mean square error,

*: p<0.01

Fig 6(e)–6(g) depict the distribution of estimation errors in the network and investigate the spatial reasons that may lead to biased evaluations. To develop a relative assessment measure, we define the absolute percentage error: |*l*_act_(*v*) − *l*_*x*_(*v*)|/*l*_act_(*v*) * 100. Nevertheless, this approach may exaggerate the error measures for the nodes that carry a low traffic load. Therefore, we define a tolerance level of ± 100 vehicles (veh) and ignore the nodes that remain within the bounds. In other words, we neglect the nodes for which the absolute difference between actual and estimated load is less than 100 veh; gray points in Fig 6(e)–6(g) represent them. For the nodes outside the tolerance bounds, we calculate the absolute percentage error. Green, yellow and red points illustrate the nodes for which the estimation error is less than 50%, between 50% and 100% and more than 100%, respectively. We note that SO produces the highest number of coloured points (especially red nodes with high error, see Fig 6(g)), while UE returns the lowest number of nodes outside the bounds and most are either green or yellow, meaning less error (see Fig 6(f)). SO conditions require more balanced flows in the network, where low usage links take over some of the excessive demand in the ‘congested’ links. The high scatter of coloured points in Fig 6(g) may be due to these low volume carrying nodes and relatively low marginal costs associated with the links around them. Additionally, the coloured points in Fig 6(e) (yellow and red points in particular) seem to compose major roadways that run along in the east-west axis, while the ones in Fig 6(f) and 6(g) are scattered around the network and do not indicate a salient pattern. This implies that free flow travel times do not accurately represent the traffic conditions on these major roads, and drivers anticipate the actual conditions on them to a certain extent and make choices accordingly. Note that in all route choice scenarios, a large portion of coloured points are located in the east of the city, in the central business district of Shenzhen, where there is a denser network of roadway links.

## Discussion

Understanding the interaction between agents in a resource limited setting has far reaching implications for many aspects of our society ranging from economic markets to social circles. In the context of traffic, the sum of all travel decisions is what defines the economic and social cost of congestion, and the coupling between travellers has fundamental importance in further steps taken to alleviate congestion.

The interaction between travel choices and traffic flows has been largely studied in the domain of traffic assignment, which forms the basis for the evaluation, assessment, design and location of transport facilities. In this work, we use sheer amount of trajectory data and investigate the empirical validity of an equilibrium state or explore the coupling between travel choices and traffic conditions. We consider three route choice scenarios based on three travel cost definitions; free flow, experienced and marginal travel time. The latter two correspond to user equilibrium and system optimum state, respectively, while the first represents a naive one-shot traffic assignment approach.

Our findings suggest contrasting results at two different scales. (i) The microscopic scale or user perspective analysis, which compares the actual and estimated paths regarding their spatial similarity, indicates no significant difference between FF and UE patterns, while they both outperform SO regarding the replication of observed routes. The similarity between FF and UE patterns is surprising, because it implies that the users do not substantially anticipate the existing traffic conditions while making route choice decisions. Additionally, even though they both outperform SO paths, neither of the patterns is able to reproduce the observed state to a large extent. (ii) The macroscopic scale or network perspective analysis focuses on the traffic loads and clearly shows that UE outperforms the others by a large margin. FF and SO produce only a moderate level of proportionality between actual and estimated node loads, and regression analysis further discloses their weakness in reproducing the observed patterns at the network scale. UE paths, on the other hand, lead to proper regression coefficients, high fitness measures and low errors, which in overall indicates the reproduction of the observed patterns to a large extent.

The main interest in the traffic assignment domain is usually to estimate the link flows and link travel demands; route or path flows hardly influence the evaluation of traffic scenarios. Further, it is well known that, while link flows are unique in the static and deterministic equilibrium, route flows are not [46]. Different assignment methods can generate different route flow output that result in the same unique link traffic flows. Our findings, indicating a strong contrast at two different scales, point at the nature of the problem which guarantees only the uniqueness of link flows.

Travellers clearly consider many alternatives when making route choice decisions, and all the observed alternatives cannot be captured by shortest paths or even advanced choice set generation algorithms [34]. However, it was not clear how the network-wide traffic patterns are affected by the mismatch of individual preferences. This paper is an attempt to fill this information gap. Despite the significant inconsistency of shortest paths at the individual level, the overall trend in the network loads is well captured by UE. Obviously, UE does not perfectly estimate the actual patterns observed in the network; our traffic load estimations do not exactly replicate the observed values. Stochastic, dynamic and bounded rationality extensions of traffic equilibrium might improve the results. This is a future research question to investigate. Further, more importantly, each of these extensions has a particular purpose that cannot be accomplished by the static and deterministic equilibrium. For instance, demand management strategies involve the manipulation and redistribution of route flows which can be estimated to a greater extent by the stochastic or boundedly rational user equilibria. And, predicting real-time traffic and developing dynamic traffic control would only be possible by the dynamic formulation of traffic equilibrium.

## Materials and methods

### Data

The data set includes GPS tracks of around 20,000 taxis in a fast growing Chinese mega-city; Shenzhen. The rapid investment created one of the fastest-growing cities in the world with a population close to 11 million and, as expected, large congestion problems both in the urban and freeway system of the city. The data set consists of trips (on the same day) from taxis equipped with a GPS sensor that stores its location every 10-40 seconds. For every GPS point, it is also known whether the taxi carries a passenger or not, which allows us to distinguish between trips with and without passengers. Assuming that taxi passengers follow routes similar to regular cars in the network, we only focus on taxi trips with passengers. We use the GPS observations from the morning peak (8AM-11AM) to produce the results presented in the manuscript, while afternoon peak (5PM-8PM) results are presented in S1 and S2 Figs.

### Road network

In this work, we apply a parser to construct routable road networks from OpenStreetMap (OSM) data. Nodes in OSM data correspond to points of interest, tags and intersections, while ways build links between nodes and include references to them. Way may contain a large number of attributes including speed limit, number of lanes, name of the street/road. Nevertheless, this information is often missing and may be inaccurate. The most consistent and accurate information that OSM provides in the road classification, including motorway, trunk, primary, secondary, tertiary, residential, trunk roads and others. In our study, as we could not find further evidence related to the application of distinct speed limits in each category of roads, we assume there are two category of roads; freeway links (consisting of motorway, trunk, primary and bridge/tunnel classes) and urban links (remaining classes including but not limited to secondary, tertiary and residential roads). Additionally, for computation purposes, we reduce the size of the network by collapsing roads (in the same road classification) with only one incoming and one outgoing road. The resulting network structure includes 10065 nodes and 23351 links.

### Map matching

In order to identify the paths that result from GPS records collected every 10-40 seconds, we apply the map-matching algorithm developed by [47] with small modifications. This procedure results in the removal of ≈20% of the available trips due to the lack of feasible paths between successive pairs of GPS records, Despite this loss, we have around 190,000 trips in the data set which offers a wide network coverage to observe traffic conditions and travel patterns. See S1 Appendix. for the details.

### Travel time estimation

In this study, we use three definitions of travel time; free flow, experienced and marginal travel time. While the calculation of free flow travel time follows a straightforward formula based on category of roads and associated speed limits, we rely on GPS records and map-matching results to estimate experienced and marginal travel times.

In order to estimate the travel cost, we formally define the road network as a directed graph *G* = (*V*, *E*) where *V* is the set of vertices or nodes, and *E* is the set of edges or links on the graph. We also denote *L* the set of link lengths associated with *E*. For each road link indexed by *e* ∈ *E*, we call *X*_*e*_ the travel time and *l*_*e*_ length of the link. Denote *N* the set of GPS observations, and for each *n* ∈ *N*, the travel time between the current and previous observation is denoted *x*_*n*_. We can represent the trajectory of observation *n* as a vector **w**_*n*_ of size *E* where $w_{n}^{e}=1$, if the edge was taken during this observation and 0 otherwise. Note that usually more than one link is crossed between two GPS points. We use three different definitions to compute travel times. The first definition is based on free flow traffic conditions;

$$\begin{array}{c} X_{e}^{ff}=l_{e}/v_{e} \end{array}$$ (3)

where we set free flow speed limit *v*_*e*_ = 100 km/h for freeways and = 40 km/h for urban roads. In the second definition, we estimate experienced travel time using the following formula:

$$\begin{array}{c} X_{e}^{exp}=(\sum_{n\in N}w_{n}^{e}\cdot l^{e})\cdot {\left(\sum_{n\in N}\frac{l_{e}}{w_{n}\cdot L}\cdot x_{n}\right)}^{-1}\;. \end{array}$$ (4)

The first term in Eq 4 refers to total distance travelled on link *e*, while the second term presents total time spent assuming vehicles have a constant speed between two successive GPS observations. Note that **w**_*n*_ ⋅ *L* defines the distance corresponding to GPS observation *n* and *l*_*e*_ / **w**_*n*_ ⋅ *L* represents the share of link *e* in terms of distance. This equation, therefore, provides an estimation of the space mean speed on link *e*.

Denoting $m^{e}=\sum_{n\in N}w_{n}^{e}$ the number of vehicles using link *e*, the third cost definition, marginal link travel time, reads as follows:

$$\begin{array}{c} X_{e}^{marg}=X_{e}^{exp}+m^{e}\cdot \frac{\partial X_{e}^{exp}}{\partial m^{e}} \end{array}$$ (5)

where the first term is the travel time experienced by an additional vehicle on the link *e* and the second term is the influence of this vehicle on the others using the same link. While $X_{e}^{exp}$ and *m*^*e*^ are easy to calculate, we do not have an analytical expression to calculate $\partial X_{e}^{exp}/\partial m^{e}$. Therefore, we follow a numeric approach here. First, we compute the link travel time experienced separately by the vehicles using link *e*, i.e. $N^{e}=\left\{n:w_{n}^{e}=1\right\}$;

$$\begin{array}{c} t_{e}^{n}=(\frac{l_{e}}{w_{n}\cdot L}\cdot x_{n})\;\forall n\in N^{e}. \end{array}$$ (6)

**Fig 7:**
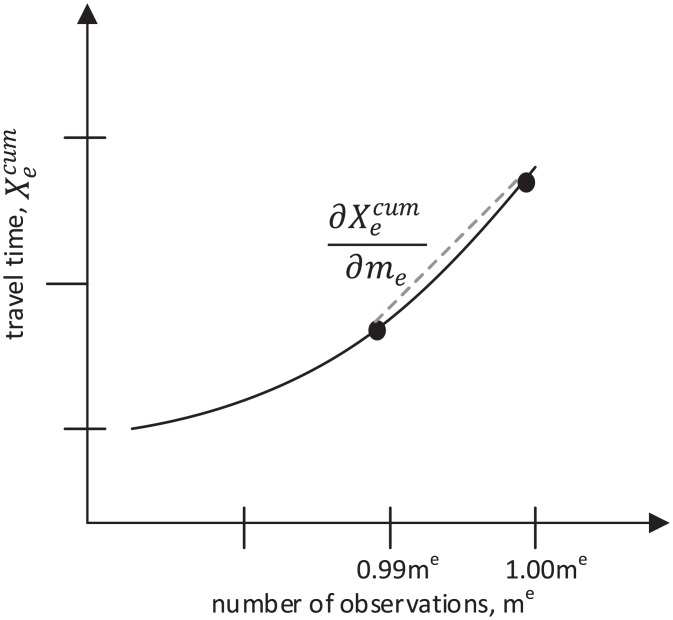
Computation of the derivative travel time.

Once we have the set of link travel times $\left\{t_{e}^{n}:n\in N^{e}\right\}$, we sort them in the ascending order, calculate the *cumulative* median of travel time for each sorted observation, i.e. $X_{e}^{cum}\left(n\right)$ (each median calculation is done for a subset from the lowest to the *n*th lowest observation), and compute the derivative using the approach illustrated in Fig 7. Note that we use the slope between 99% and 100% of the observations to compute the derivative; however, as y-axis values are the cumulative median values, our calculations are not affected by outlier observations. The derivative is defined as the slope between the last two observations in case there are less than 100 observations, and it is assumed zero if less than 10 observations. By replacing $\partial X_{e}^{exp}/\partial m^{e}$ with $\partial X_{e}^{cum}/\partial m^{e}$ in Eq 5, we can now compute the link marginal travel time. A sensitivity analysis regarding the computation of marginal travel time is provided in S2 Appendix.

## Supporting information

S1 Fig User perspective results for the afternoon peak. (EPS)

S2 Fig Network perspective results for the afternoon peak. (EPS)

S1 Appendix Map matching. (PDF)

S2 Appendix Sensitivity analysis. (PDF)

S1 File Data set. There are three files provided. 2 files are for the network description—links and nodes -, and 1 file is for the map-matched trajectory data. The raw GPS data has been downloaded from (https://www.cs.rutgers.edu/dz220/data.html).
(ZIP)
