# Supplementary material for: Searching for empirical evidence on traffic equilibrium
Source: PLoS One. 2018 May 7;13(5):e0196997. doi: 10.1371/journal.pone.0196997 (PMC5937752; doi:10.1371/journal.pone.0196997)
Supplement: S1 Appendix — (PDF) [file pone.0196997.s003.pdf]

Searching for empirical evidence on traffic equilibrium

S1 Appendix

In this study, we apply the map matching algorithm developed by [1] with small modifications. As the algorithm is originally developed for low-sampling-rate GPS trajectories, we remove/replace certain parts in order to achieve a higher performance. Essentially, the algorithm considers candidate points around each GPS observation (see Supplementary Figure 1), builds a candidate graph that considers all possible sequences (see Supplementary Figure 2) and returns the trajectory with the highest spatiotemporal matching score (see Supplementary Figure 3). The main difference we propose here is the computation of spatial and temporal scores. Following is the spatial score from the original formulation:

$$F_s(c_{i-1}^t \rightarrow c_i^j) = N(c_i^j|0,\sigma^2) \cdot \frac{dist(p_i, p_{i-1})}{path(c_{i-1}^t, c_i^j)} \tag{1}$$

where c_{i-1}^t is the tth candidate for the previous observation, c_i^j is the jth candidate for the current observation, N is the probablity density value for the standard normal distribution with variance σ^2, $dist(p_i, p_{i-1})$ is the euclidean distance between GPS observations p_i and p_{i-1} and $path(c_{i-1}^t, c_i^j)$ is the shortest path distance between candidate points c_{i-1}^t and c_i^j. While the first term indicates how likely a GPS observation can be matched with a candidate point, the second term favors the shortest paths that are similar to straight lines. This assumption is intuitively valid for two GPS points that are far apart (e.g. low-sampling-rate observations); but, as the sampling rate is 10-40s in our data set, we realized that the second term exacerbates the matching quality particularly around intersections. Therefore, removing the second term, the spatial score becomes $F_s(c_i^j) = N(c_i^j|0,\sigma^2)$.

[1] considers the cosine distance between the average speed from c_{i-1}^t to c_i^j and typical speed values along the roadway links to measure the temporal similarity. Again, bearing in mind the relatively high sampling rate in our data set, we decided to use the temporal information to filter unexpectedly high speed values. First, we calculate the average speed $\bar{v}$ and the *utmost* possible speed $\bar{v}_u$ from c_{i-1}^t to c_i^j.

$$\bar{v} = \frac{path(c_{i-1}^t, c_i^j)}{\Delta_{i-1\rightarrow i}}, \quad \bar{v}_u = \frac{\sum_{k\in p}(v_k * l_k)}{\sum_{k\in p} l_k} \tag{2}$$

where $\Delta_{i-1\rightarrow i}$ is the time difference between GPS observations, p is the sequence of links from c_{i-1}^t to c_i^j, v_k is the speed limit and l_k is the length of link k. Note $\sum_{k\in p} l_k = path(c_{i-1}^t, c_i^j)$. The temporal similarity score reads as follows.

$$F_t(c_{i-1}^t \rightarrow c_i^j) = \begin{cases} 1 & \bar{v} \leq \bar{v}_u \\ 1 - \left(\dfrac{\bar{v} - \bar{v}_u}{\alpha \cdot \bar{v}_u}\right)^2 & \bar{v}_u < \bar{v} \leq (\alpha+1)\cdot \bar{v}_u \\ 0 & \bar{v}_u < \bar{v} \end{cases} \tag{3}$$

where α is a coefficient that defines the allowed speed observation and F_t takes values between 0 and 1. Finally, the spatial similarity score is $F(c_{i-1}^t \rightarrow c_i^j) = F_s(c_i^j) * F_t(c_{i-1}^t \rightarrow c_i^j)$. For

Algorithm 1: ST Matching

Input: Road network G, a trajectory $T : p_1 \rightarrow p_2 \rightarrow ... \rightarrow p_n$
Output: The matched sequence $P : c_1^{j_1} \rightarrow c_2^{j_2} \rightarrow ... \rightarrow c_n^{j_n}$

```
Initialize tList ← ∅
foreach 1 to n do
    s = GetCandidates(p_i, G, r) // candidates within radius r
    tList.add(s) // expand the list
    G_T = ConstructGraph(tList) // build graph using all possible sequences (see
      Supplementary Figure 2)
end
return FindMatchedSequence(G_T)
```

Figure 1: Algorithm for spatio-temporal map matching

further details, please see Supplementary Figure 1-3. The parameters used in the algorithm are r=100[m], σ=20[m] and α=0.3.

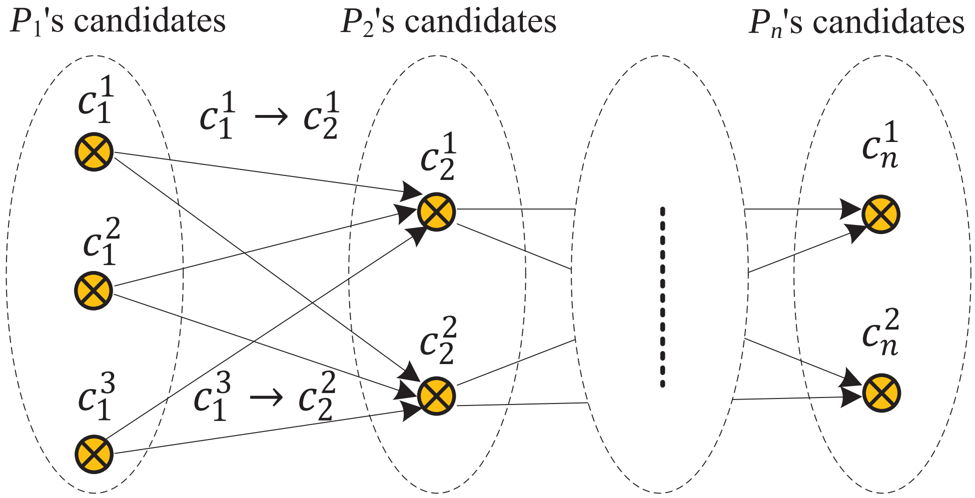

Figure 2: Candidate graph (G_T) [1]

Algorithm 2: FindMatchedSequence

Input: Candidate graph G_T
Output: The sequence with the highest score $P : c_1^{j_1} \rightarrow c_2^{j_2} \rightarrow ... \rightarrow c_n^{j_n}$

Initialize f=[] // the highest score so far
Initialize *pre*=[] // the parent of the current candidate
foreach c_1^j **do**
    $x_1^j = dist(c_1^j, p_1)$ // euclidean distance between p_1 and c_1^j
    $f[c_1^j] = N(x_1^j|0, \sigma^2)$ // observation probability based on standard normal distribution with std. dev. σ
end
foreach i=2 to n **do**
    foreach c_i^j **do**
        $max = -\infty$
        foreach c_{i-1}^t **do**
            $alt = f[c_{i-1}^t] + F(c_{i-1}^t \rightarrow c_i^j)$ // add spatiotemporal score F
            if $alt > max$ **then**
                $max = alt$
                $pre[c_i^j] = c_{i-1}^t$
            $f[c_i^j] = max$
        end
    end
end
Initialize $rList \leftarrow \emptyset$
$c = \text{argmax}_{c_n^j}(f[c_n^j])$
foreach i=n to 2 **do**
    $rList.add(c)$
    $c = pre[c]$
end
$rList.add(c)$
return *FindMatchedSequence(G_T)*

Figure 3: Algorithm for matching sequences

References

[1] Yin Lou, Chengyang Zhang, Yu Zheng, Xing Xie, Wei Wang, and Yan Huang. Map-matching for low-sampling-rate gps trajectories. In *Proceedings of the 17th ACM SIGSPATIAL international conference on advances in geographic information systems*, pages 352–361. ACM, 2009.
