# Supplementary material for: Searching for empirical evidence on traffic equilibrium
Source: PLoS One. 2018 May 7;13(5):e0196997. doi: 10.1371/journal.pone.0196997 (PMC5937752; doi:10.1371/journal.pone.0196997)
Supplement: S2 Appendix — (PDF) [file pone.0196997.s004.pdf]

Searching for empirical evidence on traffic equilibrium

S2 Appendix

To calculate the marginal travel time, we initially used the change of median travel time with respect to increasing number of vehicles. As it is not sensitive to outliers, median observation is highly used as a representative value. However, one may question the sensivity of derivative computation using the median values. Therefore, we have used percentile values ranging from 20th to 80th percentile and computed the error values and the regression coefficients that result from them. Supplementary Figure 1 introduces the sensitivity results. The values from 20th to 50th percentiles produce comparable results in terms of both error measures and regression coefficients. Nevertheless, we observe that error measures significantly increase and regression coefficients deteriorate (i.e. intercept a increasing and slope b diverging from 1) with increasing values beyond 50th percentile. In overall, considering both representative quality and the sensitivity results, we conclude that the median value (i.e. 50th percentile) is a proper measure to conduct our analysis.

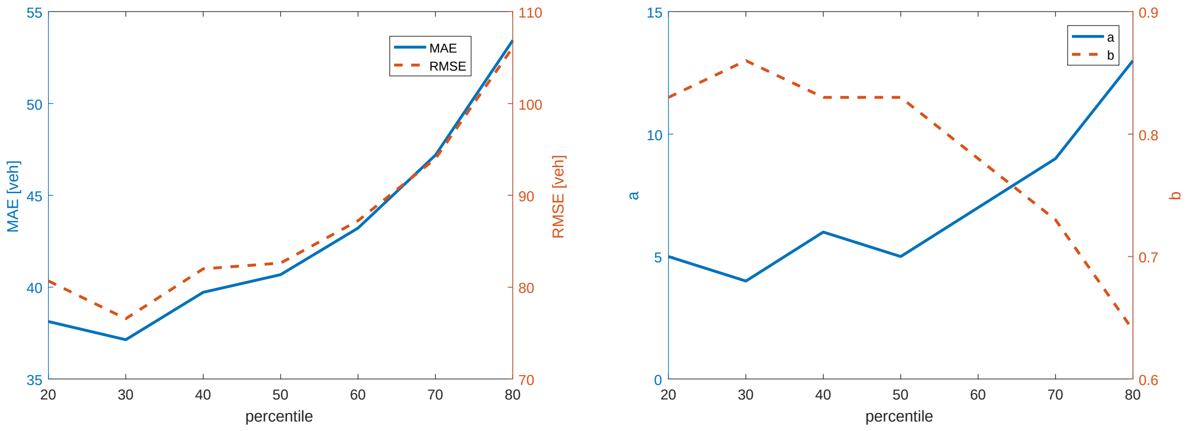

(a) (b)

Figure 1: Sensitivity of (a) error values and (b) regression coefficients to changing percentile values.
